# Design, Fabrication, and Characterisation of a Label-Free Nanosensor for Bioapplications

**DOI:** 10.3390/s22051806

**Published:** 2022-02-25

**Authors:** Mario Alberto García-Ramírez, Orfil González-Reynoso, Miguel Angel Bello-Jiménez, Everado Vargas-Rodríguez

**Affiliations:** 1 Electronics Department, Research Centre for Applied Science and Engineering, Universidad de Guadalajara, Blvd. Marcelino García Barragán 1421, Guadalajara 44430, Mexico; 2 Chemistry Engineering Department, Research Centre for Applied Science and Engineering, Universidad de Guadalajara, Blvd. Marcelino García Barragán 1421, Guadalajara 44430, Mexico; orfil.gonzalez@cucei.udg.mx; 3 Departamento de Estudios Multidisciplinarios, División Campus Irapuato-Salamanca, Universidad de Guanajuato, Av. Universidad s/n, Col. Yacatitas, Yuriria, Guanajuato 38940, Mexico; miguel.bello@uaslp.mx; 4 Instituto de Investigación en Comunicación Óptica (IICO), Universidad Autónoma de San Luis Potosí, Av. Karakorum No. 1470 Lomas 4^a^ Secc., San Luis Potosí 78210, Mexico; evr@ugto.mx

**Keywords:** biosensors, hybrid semiconductor structure, NEMS/MEMS, fabrication process, numerical analysis

## Abstract

In this paper, we present a hybrid semiconductor structure for biosensing applications that features the co-integration of nanoelectromechanical systems with the well-known metal oxide semiconductor technology. The proposed structure features an MOSFET as a readout element, and a doubly clamped beam that is isolated from the substrate by a thin air gap, as well as by a tunnel oxide layer. The beam structure is functionalised by a thin layer of biotargets, and the main aim is to detect a particular set of biomolecules, such as enzymes, bacteria, viruses, and DNA/RNA chains, among others. In here, a three-dimensional finite element analysis is performed in order to study the behaviour of the functionalised, doubly clamped beam. Preliminary results for the fabrication and characterisation processes show good agreement between the simulated and measured characteristics.

## 1. Introduction

Biosensor technology was initially described in 1965 with the development of enzyme electrodes. Since then, biosensors structures have increased in complexity, robustness, and detection sensibility. As a result, they have played a key role in providing a state-of-the-art analytical detection tool due to the inclusion of a wide variety of novel materials and techniques [1,2,3,4]. Biosensor devices have been used to detect a broad variety of bio-related targets, such as DNA/RNA chains, viruses, bacteria, and enzymes, as a function of the target used for this purpose. The identification time, cost, and versatility are the main characteristics that has put them in the front line in key security areas for diseases detection, such as airports, hospitals, and contaminated regions, where an outbreak might take place. A few approaches have been developed to use semiconductor-based structures as biosensors. Those consider polymers as the main active layer, such as SU-8 or PEG-DA, as well as complex structures, based on lab-on-a-chip structures in order to detect biomolecules [5,6,7,8]. A few others have numerically analysed larger MEMS structures based on the well-known technique of cantilevers to detect key biomolecules [9,10,11,12,13].

**Figure 1:**
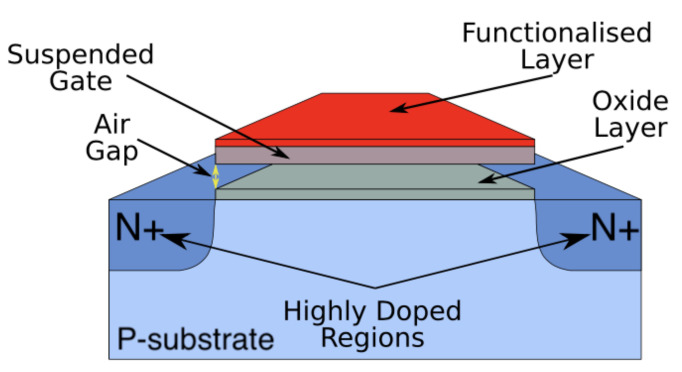
Schematic diagram of a hybrid nanostructure. The structure features a functionalised, suspended gate that is doubly isolated from the substrate by a thin oxide layer and by an air gap, and uses an MOSFET as a readout element.

In this paper, we are proposing a novel structure that co-integrates nanoelectromechanical systems with the well-known metal oxide semiconductor (MOS) technology, featuring high sensitivity and dynamic behaviour. The structure that we are proposing features an MOSFET as a readout element and a functionalised, doubly clamped beam that is isolated from the substrate by a thin oxide layer and by an air gap, as depicted in Figure 1.

The basic behaviour of the hybrid structure is as follows: When the functionalised hybrid structure is biased, the doubly clamped beam bends downwards due to the electrostatic forces that act on it. When the applied voltage is increased, the beam reaches a point called the pull-in point, after which—by a small increment on the voltage—it will collapse on the substrate. By reducing the applied voltage, the beam will remain attached beyond the pull-in point due to the static friction, produced by the electrostatic forces and the capacitor formed when collapsed. By reducing the voltage further, an imbalance between the beam stiffness and the electrostatic force will be produced and, as a consequence, the beam will be detached to return to its original isolated position.

The sensing process is performed through a dynamic movement of the functionalised suspended gate (FSG) [14,15]. When the functionalised beam detects the targeted element, it will modify the resistivity on the FSG and, as a consequence, the pull-in initial trajectory/voltage will be affected. As a result, the channel formation occurs once the beam has been collapsed on the substrate, and a current peak appears, indicating that the targeted element—such as a virus, bacteria, or DNA/RNA chain—has been found. In this paper, the influence of the short-range forces, such as the van der Waals and the Casimir forces, are analysed and implemented within a numerical analysis. By implementing those, along with the electrostatic force, within a numerical analysis, we can analyse the influence of the short-range forces in sensing applications. The paper is divided into four sections. The first section describes an introduction for biosensors and our proposal; the second section describes the the pull-in and pull-out effects due to the electrostatic and short-range forces; the third section considers a 3D numerical analysis for the pull-in/pull-out effect; finally, the fabrication process of the doubly clamped beam structure, as well as the characterisation of the SFG, are presented in Section 4.

## 2. Doubly Clamped Beam Analysis

**Figure 2:**
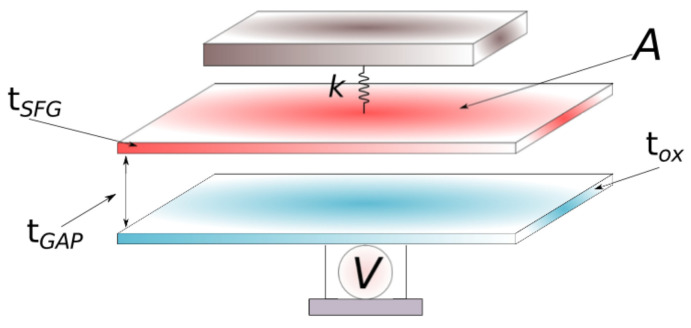
Double-plate capacitor structure model used to analyse the effects of the pull-in and pull-out effects, while a voltage is applied.

The milestone for the hybrid nanostructure relies on the functionalised suspended gate. The functionalisation process for the suspended gate is performed by depositing key receptor molecules on the surface. Those are scattered across the SG surface by keeping the size and separation to reduce the inherent parasitic molecules that might affect the selectivity of the device. In order to obtain such parameters, the Langmuir model is considered [16,17,18,19,20]. The sensing process is performed through the electromechanical, doubly clamped beam model. The model proposed to obtain the full behaviour of the suspended gate considers the use of a double-plate capacitor structure, as depicted in Figure 2. In here, the reference labels for $L_{SFG}$ and $W_{SFG}$ are the length and width of the plate capacitors, respectively; $t_{GAP}$ is the initial air gap; $t_{SFG}$ and $t_{ox}$ are the thickness for the functionalised beam and for the oxide layer, respectively; *V* is the applied voltage; and *k* is the spring constant. Here, Poisson’s ratio, Young’s modulus, and the air gap drive the pull-in voltage. By understanding the full behaviour of the functionalised gate, it is possible to improve the sensitivity of the biosensor.

*k* is defined as the material stiffness; $t_{SFG}$ and $t_{ox}$ are the thickness of the suspended functionalised gate and the oxide layer, respectively; *V* is the voltage applied; $t_{GAP}$ is defined as the initial air gap; and *A* represents the area of the plates. According to the schematic diagram shown in Figure 2, the pull-in voltage is defined as follows:

(1) $$V_{Pull-in}=\sqrt{\frac{8kt_{GAP}^{3}}{27\epsilon_{0}A}}$$

where $\epsilon_{0}$ is the space permittivity. By following a similar process, the pull-out voltage is defined as follows:

(2) $$V_{Pull-out}=\sqrt{\frac{2kt_{ox}^{2}}{\epsilon_{0}\epsilon_{ox}}\left(t_{GAP}-t_{ox}\right)}$$

where $t_{ox}$ is defined as the thickness of the oxide layer, $\epsilon_{ox}$ is the permittivity of the oxide material (SiO${}_{2}$), and $t_{SFG}$ is the initial air gap.

Due its nature, the implemented model is purely electrostatic. This model is incomplete, mainly due to the short-range forces, such as the Van der Waals and Casimir forces, which are not considered within this model [21,22]. At large ranges (micrometers), these forces are weak compared with the electrostatic force, and in most cases those are negligible for most of the analyses. By scaling down the device, the short-range forces become predominant and overcome the electrostatic force. The van der Waals and Casimir forces have strong influences on the pull-out effect rather than in the pull-in effect, that is purely electrostatic. This is why, in order to complement the pull-out equation, we added an extra term that represents the influence of the short-range forces. By rewriting Equation (2), we obtain:

(3) $$V_{Pull-out}=\sqrt{\frac{2kt_{ox}^{2}}{\epsilon_{0}\epsilon_{ox}}\left(t_{GAP}-t_{ox}\right)-\frac{A_{h}}{3\pi \epsilon_{ox}t_{ox}}}$$

where $A_{h}$ is defined as the Hamaker constant. The Hamaker constant defines the interaction among two or more materials within a media [23]. In our particular case, for the pull-out effect, it is the interaction between two materials and a media that is crucial due to high tune and sensitivity. This is the reason that the calculation of the Hamaker constant—and as a consequence the short-range forces—becomes essential. In order to calculate the Hamaker constant, a few parameters must be analysed. At the atomic level, multiple reflections are developed due to the interaction between atoms, i.e., by considering a three-atom model, where the influence of atom A induces a dipole in atom B, and, in parallel, the field of atom A also polarises atom C. The induced dipole in atom C has an influence in B. As a consequence, the field of A has a direct influence in B, and, as an effect of the reflection, C is affected. In order to surpass the additivity issue above described, a continuous theory, based on pairwise integration—that neglects the atomic structure, such as the Lifshitz theory—is used [24,25].

(4) $$A_{132}=\frac{3kT}{2}\left(\frac{\epsilon_{1}-\epsilon_{3}}{\epsilon_{1}+\epsilon_{3}}\right)\left(\frac{\epsilon_{2}-\epsilon_{3}}{\epsilon_{2}+\epsilon_{3}}\right)\cdots$$

$$\cdots \frac{3\hbar \nu_{e}}{8\sqrt{2}}\frac{\left(n_{1}^{2}-n_{2}^{2}\right)\left(n_{2}^{2}-n_{3}^{2}\right)}{\left(\sqrt{n_{1}^{2}+n_{2}^{2}}\sqrt{n_{1}^{2}+n_{2}^{3}}\right)\left(\sqrt{n_{1}^{2}+n_{2}^{2}}+\sqrt{n_{1}^{2}+n_{2}^{3}}\right)}$$

where $\nu_{e}$ is defined as the absorption frequency, and *n* and ϵ are the refractive index and the permittivity. For our analysis, we are considering the parameters described elsewhere for aluminium, air, and SiO${}_{2}$, respectively; *k* is the Boltzmann constant and *T* is the temperature in *K*.

**Figure 3:**
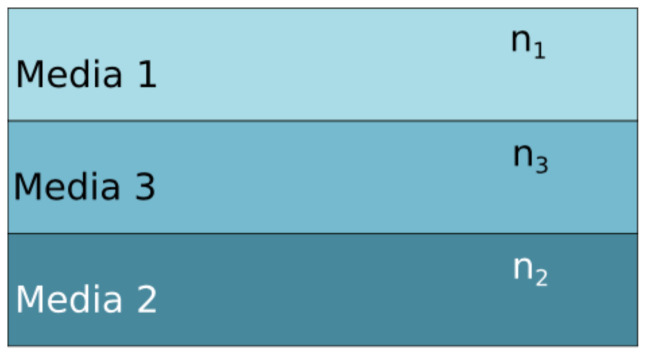
Lifshitz model implementation through three different materials, using the refractive index (n) and the permittivity (ϵ) as key features.

The model used to calculate the Hamaker constant is depicted in Figure 3.

Van de Waals and Casimir forces compared with the electrostatic force are predominant only at short range. The definition of the van der Waals forces featuring a two flat bodies is defined as follows [26,27,28]:

(5) $$F_{vdW}=\frac{A_{h}}{6\pi D^{3}}$$

where $A_{h}$ is defined as the Hamaker constant and *D* is the separation between both layers. The Casimir force for the same configuration is defined as follows:

(6) $$F_{Casimir}=\frac{\hbar c\pi^{2}}{240D^{4}}$$

where *ℏ* is the reduce Planck constant, *c* is the speed of light in the vacuum, and *D* is the initial air gap separation. The implementation of both short-range forces as part of the numerical analysis is performed by using finite element method software that allows the interaction of external forces within the analysis.

## 3. Numerical Analysis

**Figure 4:**
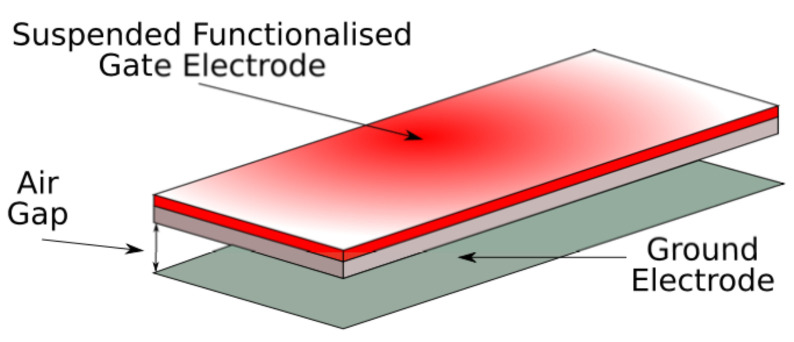
Doubly clamped beam structure used to dynamically analyse the pull-out effects through FEM-based software.

In order to analyse the behaviour of the doubly clamped beam under several forces, such as the short-range forces and the electrostatic force, a capacitor model—as sketched in Figure 4—is implemented.

To perform the numerical analysis, the parameters used are specified in Table 1.

**Figure 5:**
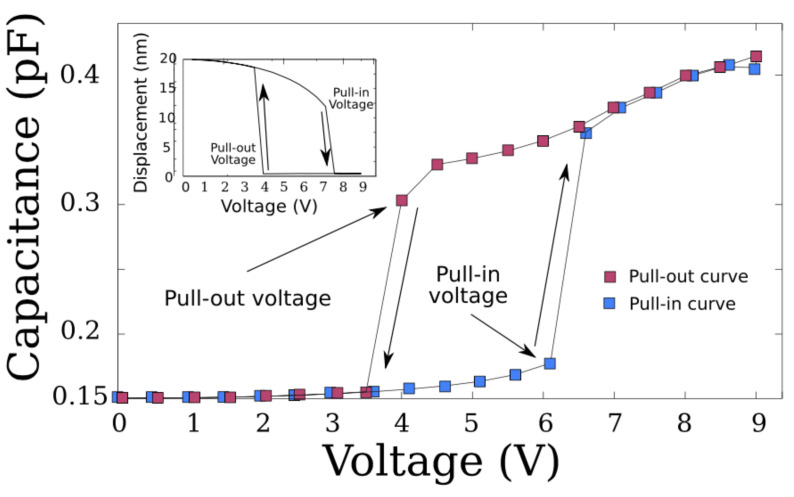
Set of curves obtained using numerical analysis. Here, the hysteresis cycle shows the trajectories of the pull-in and pull-out voltages for the double-plated capacitor model.

As a result of the analysis performed, a set of curves that describe a hysteresis cycle was obtained, as shown in Figure 5 [29,30].

**Figure 6:**
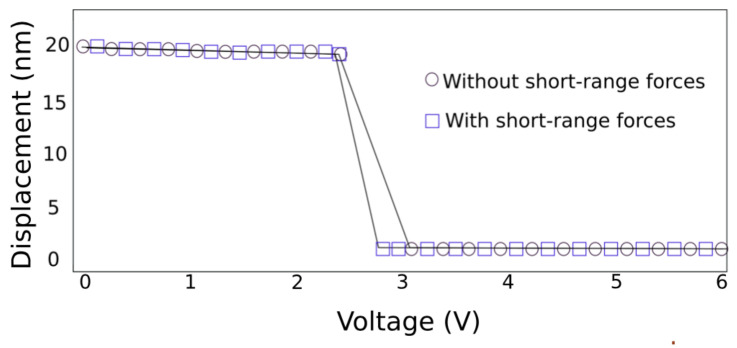
Set of pull-out trajectories featuring the electrostatic-based one (circle) in comparison with the trajectory that features the short-range forces and electrostatic forces.

The set of trajectories described in Figure 5 occurs due mainly to the electrostatic force, since the pull-in effect seems to not be affected by the quantum mechanical forces due to the large separation. In contrast, for the pull-out effect, it is mainly governed by the short-range forces rather than by the electrostatic force. Figure 6 shows how the van der Waals and the Casimir forces act on the pull-out effect. The pull-out voltage was found to be reduced by 300 mV due to the interaction of the aluminium doubly clamped beam (≈70 GPa) with the short-range forces.

## 4. Hybrid Structure Fabrication

**Figure 7:**
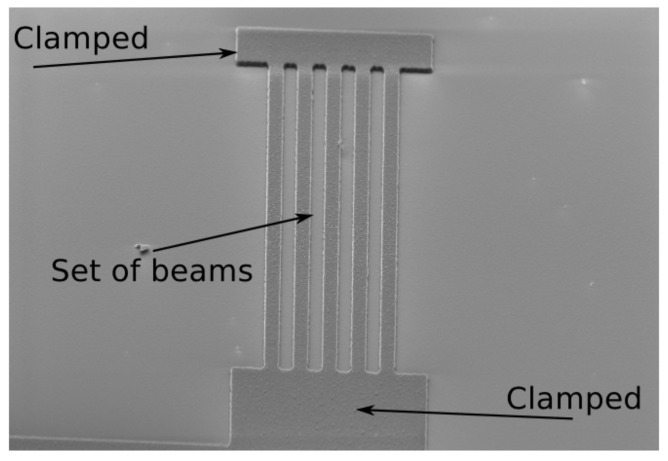
SEM image that shows a set of doubly clamped beams after the wet-etching process.

To fabricate the hybrid structure, we developed a fabrication process in collaboration with the Nanofab of the University of Southampton. We deposited 20 nm of SiO${}_{2}$ on a thick silicon substrate. As a sacrificial layer, we used 200 nm of Poly–Si and an aluminium layer 500 nm thick was deposited on top of it. In order to pattern the doubly clamped beam shapes, a high-resolution resist was used, such as the S1813, that was used under UV light. Once processed, the wafer was rinsed in developer for 60 s and rinsed in DI water for 120 s. In order to remove the aluminium excess and shape the beams, the sample is first etched by using aluminium etcher for 240 s at 300 K, as a result of the patterning process—the patterned beam is depicted in Figure 7.

**Figure 8:**
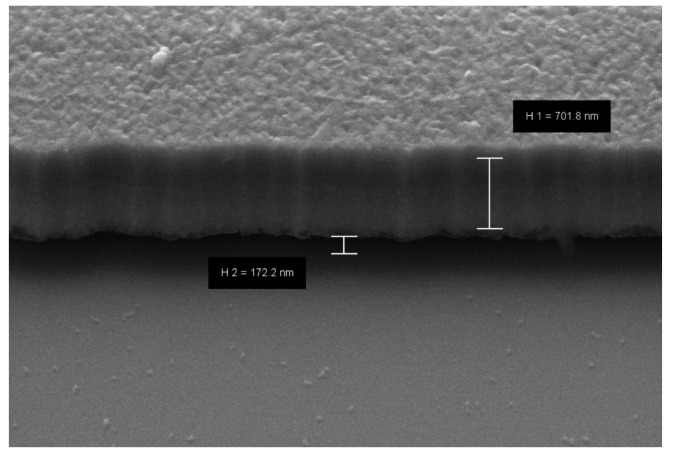
SEM image that displays the the cross-section of the beam successfully suspended, once the dry-etching process was performed.

After performing this step, the sample was rinsed with DI water and dried by N${}_{2}$. In order to suspend the beams, the sacrificial layer was removed using a plasma etching process, using SF${}_{6}$ and O${}_{2}$ as working gases. As a result of this process, we successfully obtained a suspended, double-clamped beam. Figure 8 shows an SEM cross-section image of the suspended beams.

**Figure 9:**
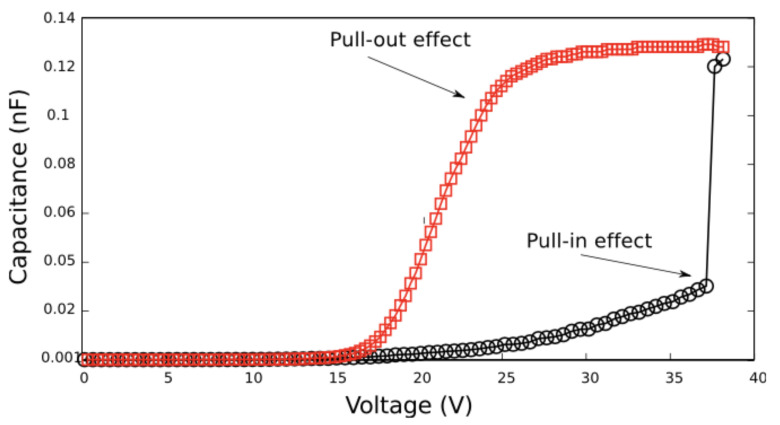
Hysteresis cycle obtained for the characterisation of the functionalised suspended beam. The pull-in effect was obtained as the numerical analysis shows. In contrast, the pull-out effect occurred in a smooth way due to the length of the beam and because of the short-range forces.

As a result of the fabrication process, we proceeded to characterise the hybrid structure. We found that the pull-in effect for a long FSG took place, as shown in Figure 9. Nevertheless, the pull-out effect obtained from the hybrid structure was smooth due to the stiffness of the material used.

For this application, the detachment process is required to be fast. This is why, we performed further numerical analysis in order to find the stiffness value that is capable to surpass such set of forces. As a result, it was found that the stiffness for the doubly clamped beam material needs to be higher than 160 GPa. According to the literature, a material that presents such stiffness is poly-Si. This material is capable of surpassing the electrostatic and the short-range forces once the applied voltage has been reduced beyond the pull-in voltage.

## 5. Conclusions

In this paper, we presented a hybrid nanostructure for biosensing applications that features the co-integration of nanoelectromechanical systems with the well-known metal oxide semiconductor technology. The structure featured an MOSFET as a readout element and a doubly clamped beam, that is isolated from the substrate by a thin air gap as well as by a tunnel oxide layer. The beam structure was functionalised by a thin layer of antigens that aims to detect a particular set of biotargets, such as viruses, bacteria, enzymes, and DNA/RNA chains. A three-dimensional finite element analysis was performed in order to study the behaviour of the doubly clamped functionalised beam. Preliminary results showed good agreement between the simulated and measured characteristics, as well as key capabilities of the nanostructure to be used in the detection of a broad variety of biomolecules.

## Figures and Tables

**Table 1:** Set of parameters used to numerically analyse the pull-in and pull-out voltages.

Material	Thickness (nm)	Layer
Aluminium	30	SG
Air	20	Gap
SiO${}_{2}$	7	Tunnel Oxide
Si	100	Mechanical Support
